# A meeting to celebrate the centennial birthday of Yuan-Cheng Fung: the father of modern biomechanics and foreign member of the Chinese Academy of Sciences

**DOI:** 10.1093/nsr/nwz151

**Published:** 2020-02-04

**Authors:** Yi Cao

**Affiliations:** Department of Physics, Nanjing University

On 20–23 September 2019, many students, friends and colleagues of Professor Yuan-Cheng Fung, together with more than 120 scientists and engineers worldwide, gathered at Catamaran Resort in San Diego to attend the first International Conference on Biomechanics and Medical Engineering (ICBME2019) and celebrate the 100th birthday of Fung, the father of modern biomechanics and the creator of tissue engineering.

Professor Shu Chien, a member of the National Academy of Sciences of the USA and a foreign member of the Chinese Academy of Sciences, served as the honorary chair of ICBME2019. Professor Andrew D. McCulloch of the University of California, San Diego, Professor Jay D. Humphrey of Yale University, Professor Roger D. Kamm of the Massachusetts Institute of Technology, and Professor Dalin Tang of Worcester Polytechnic Institute served as co-chairs of the conference.

The conference started with Professor Chien’s talk about Professor Fung’s career and scientific achievements. He introduced Professor Fung’s remarkable findings on residual stress in arteries, the stress–strain relationship of living tissues, the effect of mechanical stress on remodeling, growth and resorption of tissues, the theory of sheet flow in lung alveoli, determination of RBC 3D geometry by interference microscopy, the growth and remodeling of blood vessels under stress in health and disease, and the invention of ‘Biodyne’ for testing the mechanical properties of biological materials—just to mention a few. Fung’s research had led to enormous prestigious awards and honors, including the Founders Award from the US National Academy of Engineering and the US National Medal of Science. He shared many pictures of Professor Fung with his family, friends and colleagues. He also showed Professor Fung’s beautiful drawing and carved seals. In his mind, Professor Fung is a renaissance man, who is not only a great scientist and engineer, but also an artist.

Professor Fung’s scientific achievements were further commented on by Professor Humphrey in his talk. The book entitled *Elasticity of Soft Tissues in Simple Elongation* written by Professor Fung has been cited more than 10 000 times. More impressively, his highly cited papers on the residual stresses in blood vessels and the indicial functions of arterial remodeling were published when he was 72 and 77 years old, respectively. Professor Fung dedicated himself to biomechanics studies even in his old age. He has become a role model for many scientists in this field.

**Figure 1:**
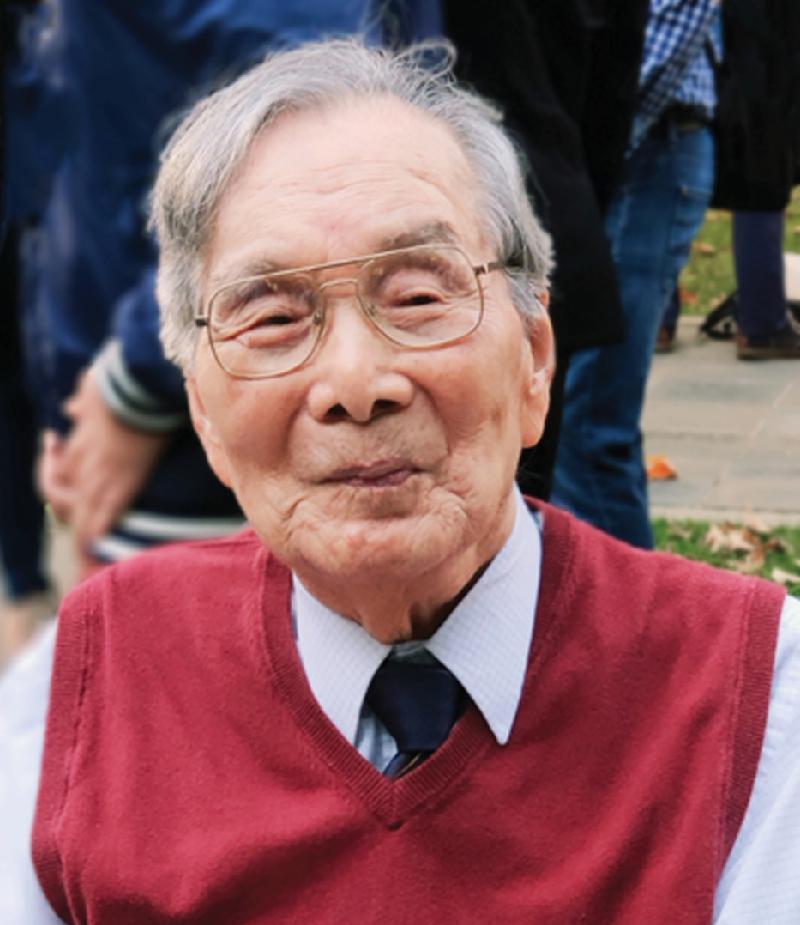
A picture of Professor Yuan-Cheng Fung taken on his 100th birthday celebration. (*Courtesy of Yi Cao*)

Professor Savio L-Y. Woo from the University of Pittsburgh further gave a complete summary of Professor Fung’s influential roles to the whole scientific community and to himself. In his mind, Professor Fung is ‘a consummate teacher, a distinguished scholar, a brilliant researcher, a quintessential leader, a visionary role model, and more personally, a revered mentor, a generous colleague, and a loving friend’. He also called Professor Fung a 4G person: Genius, Gentle, Genuine and Generous. Although Professor Woo is a world-renowned bioengineer and member of the National Academy of Engineering of the USA, he humbly called himself a follower and admirer of Professor Fung.

Other younger speakers have frequently mentioned their connections or interactions with Professor Fung in their academic careers. They considered Professor Fung a respected mentor and beloved friend. Obviously, Professor Fung has successfully inspired, taught and guided many junior colleagues in the field of biomechanics.

All conference participants got a chance to meet Professor Fung face to face and say ‘Happy birthday’ to him in the elegant Y. C. Fung Auditorium of the Powell-Focht Bioengineering Hall on the University of California, San Diego campus. It was such a great opportunity and no one wanted to miss this event. Numerous photos were taken to record this unforgettable time! Professor Fung always kept a gentle smile on his face while taking photos with many friends, colleagues and junior researchers. Obviously, he is still influencing the biomechanics field, which he had pioneered and led for many years.

This conference was sponsored by Tech Science Press, Department of Bioengineering, the University of California, San Diego, Key Laboratory of Bio-Rheology Science and Technology, Chongqing University, Shanghai Biophysical Society and the Jiangsu Society of Theoretical and Applied Mechanics.

*Professor Yuan-Cheng Fung passed away recently at the UCSD Jacobs Hospital. We mourn deeply the tragic loss of such a scientific giant, wonderful colleague, and magnificent mentor. His character and his contributions shall forever be remembered.*

